# Neuronal-Specific Deficiency of the Splicing Factor *Tra2b* Causes Apoptosis in Neurogenic Areas of the Developing Mouse Brain

**DOI:** 10.1371/journal.pone.0089020

**Published:** 2014-02-19

**Authors:** Markus Storbeck, Kristina Hupperich, John Antonydas Gaspar, Kesavan Meganathan, Lilian Martínez Carrera, Radu Wirth, Agapios Sachinidis, Brunhilde Wirth

**Affiliations:** 1 Institute of Human Genetics, University of Cologne, Cologne, Germany; 2 Institute of Genetics, University of Cologne, Cologne, Germany; 3 Center for Molecular Medicine, University of Cologne, Cologne, Germany; 4 Insitute of Neurophysiology, University of Cologne, Cologne, Germany; Duke University Medical Center, United States of America

## Abstract

Alternative splicing (AS) increases the informational content of the genome and is more prevalent in the brain than in any other tissue. The splicing factor *Tra2b* (*Sfrs10*) can modulate splicing inclusion of exons by specifically detecting GAA-rich binding motifs and its absence causes early embryonic lethality in mice. *TRA2B* has been shown to be involved in splicing processes of *Nasp* (nuclear autoantigenic sperm protein), *MAPT* (microtubule associated protein tau) and *SMN* (survival motor neuron), and is therefore implicated in spermatogenesis and neurological diseases like Alzheimer’s disease, dementia, Parkinson’s disease and spinal muscular atrophy. Here we generated a *neuronal*-specific *Tra2b* knock-out mouse that lacks *Tra2b* expression in neuronal and glial precursor cells by using the *Nestin-Cre*. Neuronal-specific *Tra2b* knock-out mice die immediately after birth and show severe abnormalities in cortical development, which are caused by massive apoptotic events in the ventricular layers of the cortex, demonstrating a pivotal role of Tra2b for the developing central nervous system. Using whole brain RNA on exon arrays we identified differentially expressed alternative exons of *Tubulinδ1* and *Shugoshin-like2* as *in vivo* targets of Tra2b. Most interestingly, we found increased expression of the cyclin dependent kinase inhibitor 1a (p21) which we could functionally link to neuronal precursor cells in the affected brain regions. We provide further evidence that the absence of *Tra2b* causes *p21* upregulation and ultimately cell death in NSC34 neuronal-like cells. These findings demonstrate that *Tra2b* regulates splicing events essential for maintaining neuronal viability during development. Apoptotic events triggered via *p21* might not be restricted to the developing brain but could possibly be generalized to the whole organism and explain early embryonic lethality in *Tra2b*-depleted mice.

## Introduction

Alternative splicing increases the informational content of the genome allowing a relatively small number of genes to encode a much larger number of mRNAs and proteins [1]. Virtually all transcripts encoded by multiple exons undergo alternative splicing and numerous splicing events are evolutionarily highly conserved [2]. Alternative splicing can modify protein function by adding or exchanging coding information and might target a transcript for nonsense-mediated decay (NMD) by insertion of premature termination codons (PTCs) [1], [3], [4]. Exon inclusion is based on splice site selection by the spliceosome, an assembly of small nuclear ribonucleic particles (snRNPs), SF1, U2AF and numerous other factors that facilitate the removal of introns from primary transcripts [5], [6]. Splice site selection by the spliceosome is modified by a great number of trans-factors that bind to exonic as well as intronic sequence motives, which may promote either exclusion or inclusion of an exon [7]. This makes exon recognition a subject of complex regulatory mechanisms including intrinsic splice site strength, the number and position of enhancing and silencing elements as well as RNA secondary structure. Altogether this conveys significant flexibility to spliceosomal exon recognition [8], [9].

The splicing factor TRA2B (SFRS10, ENSG00000136527, ENSMUSG00000022858) belongs to the family of arginine−/serine-rich splicing factors and contributes to alternative splicing of specific exons. The Tra2 protein specifically targets exons via (A)GAA binding motifs, thereby promoting their splicing inclusion [10], [11], [12]. However, it has been reported that Tra2b can promote exon skipping dependent on its binding position [13], [14], [15] or when acting indirectly through other splicing factors independently of its RNA recognition motif (RRM) [16], [17].

The absence of the *Tra2b* paralog *transfromer-2* in *D. melanogaster* causes sex reversal due to altered splicing of the *dsx* transcript [18], [19], [20] and mice ubiquitously depleted of *Tra2b* die at early gestational stages, suggesting a fundamental role in development [21]. Specific *TRA2B*-related splicing processes have been implicated in human diseases like spinal muscular atrophy (splicing of *SMN*) [22], [23], [24] and in tauopathies like Alzheimer’s disease and FTDP-17 (splicing of *MAPT*) [25], [26], [27], [28]. Moreover, *TRA2B* is reported to be involved in neuronal function (splicing of *CLTB*) [15], [29], [30], vascular smooth muscle diversification (splicing of *MYPT*) [31] and in obesity by regulation of lipogenesis (splicing of *LPIN1*) [32].

Previously, we reported that the T-exon of the histone chaperone *Nasp* (nuclear autoantigenic sperm protein, ENSMUSG00000028693) is spliced by Tra2b in mouse testis and embryonic brain [17]. Tra2b recognizes and directly binds the *Nasp* T-exon via a high number of clustered and evolutionarily conserved GAA-rich motives to promote its inclusion. *Nasp* encodes two isoforms: the somatic isoform *sNasp* and the testis-enriched isoform *tNasp*, which comprises a roughly 1 kb long T-exon [33]. Besides its high expression in testis *tNasp* is reported to be highly abundant in embryonic and tumor cells, which are both highly proliferative cell types [33], [34]. O’Rand and colleagues have demonstrated that selective depletion of *tNasp* in tumor cells abolished proliferation [35] and ablation of *Nasp* causes early embryonic lethality in mice [34]. This data implicates *Nasp* playing a fundamental role during embryonic development and probably in highly proliferative cells in general.

Histone deacetylase inhibitors (HDACi) are substances inhibiting HDAC function, thus attenuating chromatin condensation and generally enhancing gene expression. HDAC inhibitors like valproic acid and M344 have both been demonstrated to transcriptionally upregulate *Tra2b* [24], [36]. Valproic acid is used for the therapy of epilepsy [37], bipolar disorder [38], migraine [39] as well as spinal muscular atrophy (SMA) [24] and anti-cancer effects have been attributed to VPA due to the transcriptional upregulation of tumor suppressor genes [40], [41], [42]. The effect of *Tra2b* upregulation by VPA is exploited in the treatment of SMA, where it promotes splicing correction of the *SMN2* exon 7 [24], [36]. However, increased Tra2b concentrations likely affect the splicing of various exons and the consequential deregulation of splicing could cause side effects during HDACi treatment. Here we sought to identify novel Tra2b splicing targets *in vivo* to determine putative splicing-related off-targets in treatment strategies involving HDACi. In the brain there is a higher occurrence of alternative splicing events than in any other tissue [43], [44], [45] and 50 percent of known debilitating mutations in RNA-binding proteins cause neuronal-related diseases [46], [47]. Given the massive involvement of *TRA2B* during development [21] and in numerous neurological conditions, we generated a neuronal-specific *Tra2b* knock-out mouse to identify novel splicing targets of Tra2b in the central nervous system. Our knock-out mice express *Cre-recombinase* under the control of the rat *nestin* promoter [48] and are depleted of *Tra2b* in neuronal and glial precursors.

Here we demonstrate that *Tra2b* is essential for neuronal development and survival and identify exons of Shugoshin-like 2 (*Sgol2,* ENSMUSG00000026039) and Tubulin delta chain (*Tubd1,* ENSMUSG00000020513) as splicing targets of Tra2b using mouse whole exome array analysis. We provide evidence that in absence of *Tra2b* neuronal development is disturbed by p21-mediated cell cycle inhibition resulting in apoptosis and a loss of proliferative potential in neurogenic areas of the brain and in a neuronal precursor cell culture system. We speculate that Tra2b-mediated splicing of *Nasp* might be functionally related to neuronal development and expression of the *tNasp* isoform might be necessary to preserve cell viability in the developing central nervous system.

## Materials and Methods

### Ethics Statement

All procedures involving laboratory animals were performed according to the German laws of animal welfare and were approved by the ‘Landesamt fuer Natur, Umwelt und Verbraucherschutz NRW’ under the reference numbers 50.203.2-K 38, 19/05 and 8.87-51.05.20.10.106.

### Generation of Neuronal-specific *Tra2b* Knock-out Mice and Mouse Handling

Neuronal-specific *Tra2b* knock-out mice were generated by cross-breeding *Tra2b^fl/fl^* mice [21] with *Tra2b^fl/+^; Nestin-Cre^tg/0^* mice. All mice were bread on a >96% C57BL/6N background. *Nestin-Cre* transgenic mice express *Cre-recombinase* under control of the rat *Nestin* promoter and enhancer and enable expression of *Cre-recombinase* in neuronal and glial precursors [48], [49], [50]. In a GFP reporter-line *Nestin* has been shown to be expressed in the ventricular and subventricular zone of the cortex [50]. For timed breedings the mating was allowed over night and females were checked for vaginal plugs in the morning. The day of plug detection was defined as 0.5 days postcoitum (dpc). For genotyping DNA was isolated from tail tip biopsies according to standard protocols and PCR using established conditions [21] (**Dataset S4**). For embryo or tissue preparation, euthanasia was performed by slowly increasing the carbon dioxide concentration in the animal housing.

### (Immuno-)Histochemistry

Mouse embryos were isolated from pregnant females at indicated times by standard dissection protocols in PBS. Embryonic brains were fixated in 4% paraformaldehyde/PBS over night, dehydrated by immersion in ethanol series and embedded in paraffin. Histological stainings were performed according to standard protocols. In short, paraffin-embedded sections were deparaffinized in xylene, rehydrated by immersion in ethanol-series and shortly washed in PBS and water. For hematoxylin and eosin staining we used Mayer’s hematoxylin solution and Accustain eosin y solution (Sigma-Aldrich, Munich, Germany). Sections were incubated for 5 minutes in hematoxylin, washed for 15 minutes in water and stained for 30 seconds in eosin. For immunohistological stainings the rehydrated sections were subjected to antigen retrieval by boiling 3 times for 5 minutes in 0.01 M citrate buffer (pH 6). Sections were blocked for 45 minutes at room temperature in 20% serum and 1% BSA in TBS. Primary antibodies were applied over night at 4°C in TBS with 3% milk powder. For specific detection we used the following primary antibodies: rabbit-anti-Tra2b [23] 1∶8,000; rabbit-anti-Caspase-3 (R&D Systems, Minneapolis, USA) 1∶1,000; rabbit-anti-Ki67 (Abcam, Cambridge, UK) 1∶1,000. For detection we used a biotinylated anti-rabbit secondary antibody (1∶2,000 in TBS with 3% milk powder) for 30 minutes at 4°C and the vectastain ABC kit according to the manufacturer’s instructions (Vector Laboratories, Burlingame, USA). For colorimetric detection the DAB peroxidase substrate kit (Vector Laboratories, Burlingame, USA) was used. Images were recorded on a Zeiss Axio microscope and the Zeiss M2 imaging system (Zeiss, Jena, Germany).

### Mouse Exome Microarrays

Mouse exon arrays were performed in the laboratories of Prof. A. Sachinidis at the Institute of Neurophysiology at the University of Cologne. Whole brain RNA samples of 16.5 dpc *Tra2b^fl/+^* and *Tra2b^fl/fl^; Nestin-Cre^tg/o^* mice were prepared using the Ambion WT Expression Kit (Ambion,Darmstadt, Germany) to amplify and generate sense strand cDNA with unbiased coverage of the transcriptome. cDNA was labeled and prepared using the GeneChip WT Terminal Labeling and Hybridization Kit (Affymetrix, Santa Clara, CA, USA). The array used was GeneChip Mouse Exon 1.0 ST Array (Affymetrix, Santa Clara, CA, USA). This array covers approximately 1 million exon clusters and 1.4 million probesets. Both gene level and exon level analysis were performed using *R*, Bioconductor package and Partek Genomic Suite tools. Preprocessing of exon array data included background correction using RMA [51], adjusting for GC-content, Quantile normalization and probeset summarization using mean and setting log probes using base 2. The probeset class, Gene extended was used. Preprocessing was performed by Partek Genomic Suite. Gene level analysis was performed in core extended mode. Transcripts showing very low expression signal values of less than 25 across arrays were eliminated and only 23,331 transcripts (annotated and non-annotated) were taken for statistical analysis. Moderated t statistics of linear model analysis [52] was employed to filter the differentially expressed transcripts. Threshold values of t score-p value of ≤0.05 and fold-change value 1.5 were used to generate a significant gene list. Partek Genomic Suite was used for statistical filtering of the alternatively spliced variants. Prior to alternative splicing calculation the probe sets with signals <3 (at log 2 scale) were excluded. The threshold values used for statistical filtering were set to p-value ≤0.05 and fold-change values of 1.5 for high confidence. Data obtained from array analyses are available at http://www.ebi.ac.uk/arrayexpress/with the accession number E-MTAB-2157.

### Quantitative and Standard Polymerase Chain Reaction

DNA isolation from tail tip biopsies was carried out by standard protocols or using the Qiagen DNA Blood and Tissue Kit (Qiagen, Hilden, Germany). RNA was isolated from cells and tissues using the RNeasy Mini Kit and from fatty tissues using the RNeasy Lipid Tissue Mini Kit (Qiagen, Hilden, Germany). For all RNA isolations DNAse digestion was routinely performed. Concentrations were determined using a NanoDrop spectrophotometer (Peqlab, Erlangen, Germany) and the Quant-iT RiboGreen RNA reagent and kit (Life Technologies, Darmstadt, Germany). Reverse Transcription was performed using the QuantiTect Reverse Transcription Kit (Qiagen, Hilden, Germany). For routine PCR we used recombinant Taq DNA polymerase and for cloning procedures AccuPrime Pfx DNA polymerase (Life Technologies, Darmstadt, Germany). For quantitative real-time PCR we used LightCycler FastStart DNA Master SYBR Green I on a Roche Light Cycler 1.5 (Roche, Rotkreuz, Switzerland). Oligonucleotides and PCR conditions are given in **dataset S4**.

### Minigene Splicing Assay

To assess splicing events we used the pSPL3-based exon trapping system from Life Technologies (Darmstadt, Germany). In short, the genomic region of the exon of interest with at least 500 bp of flanking intronic sequence was amplified from gDNA by PCR and cloned into the pSPL3 vector. Oligonucleotides are given in **dataset S4**. The vector contains a portion of the HIV-1 *tat* gene, an intron and splice donor and acceptor sites. The genomic region with the exon of interest is cloned into the intron, which is located in between the *tat* exons. The vector was subsequently co-transfected into HEK293T cells using the DharmaFect I transfection reagent (Thermo Scientific, Schwerte, Germany). Dependent on splice site detection the mRNA produced from the vector does either contain only the *tat* exons or additionally includes the exon of interest. The amount of each mRNA was determined by semi-quantitative RT-PCR. The splicing assay was performed while overexpressing *GFP-TRA2B* from pEGFP [23] or silencing *Tra2b* using specific siRNAs (Qiagen, Hilden, Germany).

### Cell Culture

Murine embryonic fibroblasts were generated from 13.5 dpc embryos. Embryos were isolated from pregnant females by standard dissection protocols in PBS. Material was ground and washed through a 70 µm nylon filter with serum complemented DMEM. Cells were plated and incubated at 37°C, 5% CO_2_ and 95% relative humidity. To block nonsense-mediated decay for detection of splicing isoforms cells were treated with 100 µg/ml emetine for 10 hours. *Tra2b* expression was depleted by stable transfection of a *Cre-recombinase-GFP* expressing vector into cells with the genotype *Tra2b^fl/fl^*. *Cre-recombinase* was subcloned from pTriEx-1 kindly provided by Dr. Frank Edenhofer [53]. MEFs were transfected using the Mirus TransIT-LT1 transfection reagent (Madison, WI, USA) and grown under selective conditions.

Effects on tNasp expression were investigated in NSC34 cells [54]. NSC34 cells were a kind gift from Dr. Neil R. Cashman (Totonto) and have been produced by fusion of **n**euroblastoma cells with embryonic **s**pinal **c**ord cells. NSC34 cells display a neuron-like phenotype and are considered to resemble many aspects of motoneuron development [54]. HEK-293T cells [55] (human embryonic kidney) were obtained from ATCC. HEK-293T cells and NSC34 cells were transfected with plasmid DNA and/or siRNA using the DharmaFect1 Transfection Reagent (Thermo Scientific, Schwerte, Germany). Cells were harvested after 24 h, 48 h or 72 h for isolation of RNA and after 48 h and 72 h for the isolation of proteins. Cells harvested 72 h after transfection, were re-transfected after 48 h using initial conditions to maintain sufficient expression from a vector or sufficient knock-down from an siRNA, respectively.

### Western Blot

Protein lysates from cells and tissue were prepared according to standard procedures. Samples were subjected to SDS-PAGE, transferred to PVDF blotting membranes and blocked for 2 hours at 4°C in TBST with 5% milk powder. For immunological detection of Tra2b we used rabbit-anti-Tra2b antibody 1∶2,000 in TBST with 2% milk powder over night [23]. Cdkn1a protein was detected by rabbit-anti-p21 (ProteinTech, Chicago, USA) 1∶1,000 in TBST with 2% milk powder over night. All quantification was normalized to beta-actin using mouse-anti-β-actin (Sigma-Aldrich, Munich, Germany) 1∶30,000 in TBST with 2% milk powder for 2 hours. For detection we used the horseradish-coupled secondary antibodies goat-anti-mouse-HRP (Jackson ImmunoResearch, Suffolk, UK) and goat-anti-rabbit-HRP (Thermo Scientific, Schwerte, Germany). As substrate for detection we used SuperSignal West Pico Chemiluminescent Substrate (Thermo Scientific, Schwerte, Germany).

## Results

### Homozygous Loss of *Tra2b* in Neuronal and Glial Precursor Cells of the CNS Causes Perinatal Lethality in Mice

**Figure 1:**
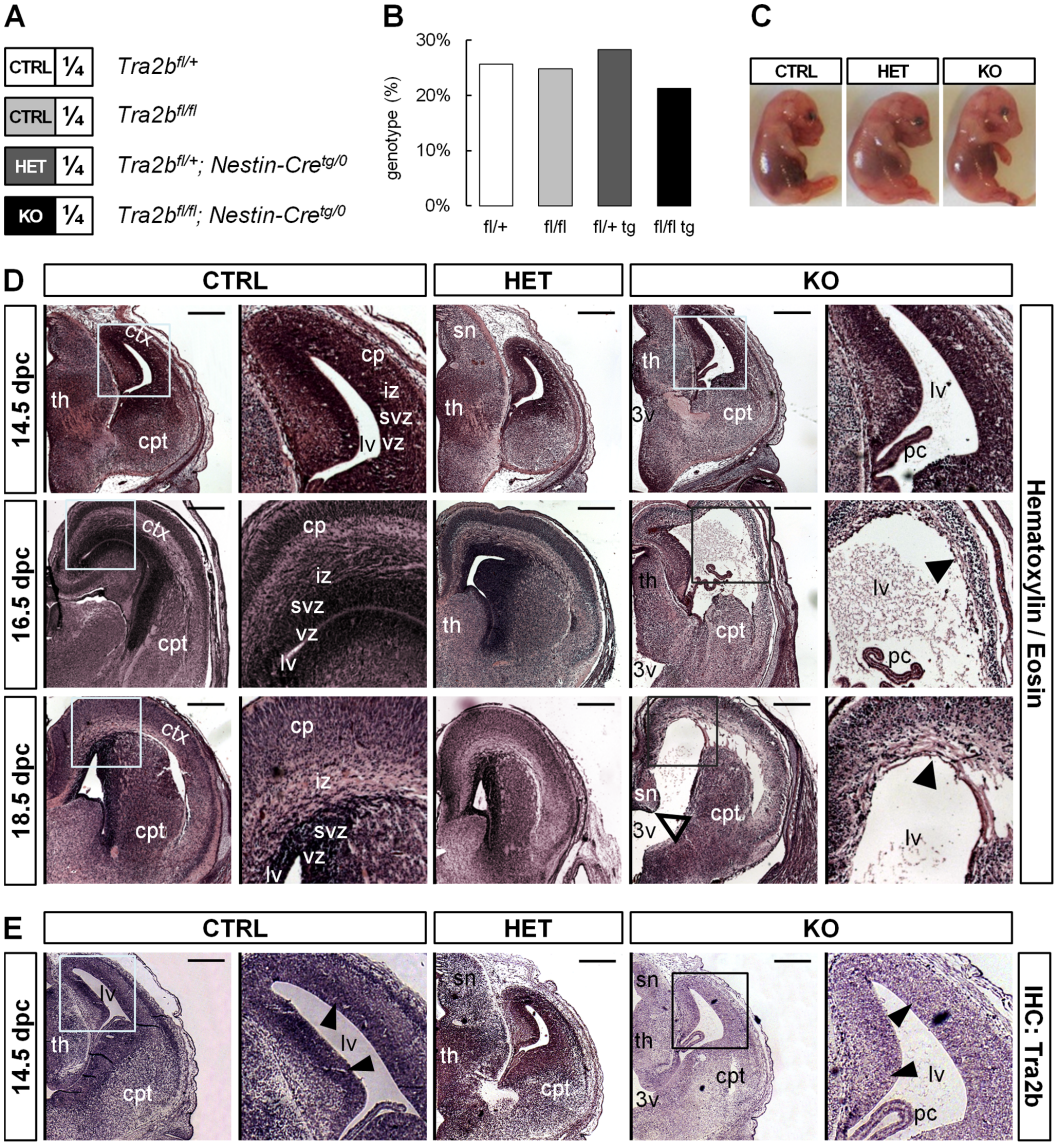
Conditional ablation of *Tra2b* causes perinatal lethality and disturbed cortical patterning in mice. (**A**) Cross breading of *Tra2b^fl/fl^* with *Tra2b^fl/+^; Nestin-Cre^tg/0^* mice allowed generation of neuronal-specific knock-out (KO) animals as well as controls (CRTL) and heterozygous knock-out animals (HET) in one litter. (**B**) KO animals are born alive and all possible genotypes were detected according to Mendelian law (N = 113). (**C**) General development of conditional knock-out mice is not impaired as there are no gross morphological differences in embryo appearance. (**D**) Hematoxylin/Eosin staining of paraffin-embedded coronal sections at indicated developmental stages. KO animals but not controls or HET animals show ventriculomegaly of the third and lateral ventricles starting at around 14.5 dpc. Cortical layers are largely distinguishable at 14.5 dpc but cortical patterning and the ependymal lining of the lateral ventricle appears highly disturbed (black arrowheads) at 16.5 dpc in knock-out brains. (**E**) Immunostaining of *Tra2b* on paraffin-embedded coronal sections shows efficient downregulation of *Tra2b* protein in knock-out brains compared to controls and heterozygote animals. Cells of the ventricular and subventricular zones of the cortex show strongest decrease in staining intensity (black arrowhead). Scale bar equals 400 µm; ctx, cortex; th, thalamus; cpt, caudoputamen; cp, cortical plate; iz, intermediate zone; svz, subventricular zone; vz, ventricular zone; sn, septal nuclei; 3v, third ventricle; lv, lateral ventricle; pc, choroid plexus.

To generate neuronal-specific knock-out mice we crossbred homozygously floxed *Tra2b* mice (*Tra2b^fl/fl^*) [21] with heterozygously floxed *Tra2b* mice carrying the *Nestin-Cre* transgene (*Tra2b^fl/+^; Nestin-Cre^tg/0^*). This *Cre* transgene is expressed under control of the rat *Nestin* promoter and enhancer [48]. Nestin expression is first activated at 10.5 dpc in the neural tube, the somites and in migrating cells of the neural crest. At 15.5 dpc it has been reported to be strongly expressed in the ventricular and subventricular zone of the cortex [49]. Using this breeding control mice (CTRL, *Tra2b^fl/+^ and Tra2b^fl/fl^*), heterozygous neuronal-specific knock-out mice (HET, *Tra2b^fl/+^; Nestin-Cre^tg/0^*) and homozygous neuronal-specific knock-out mice (KO, *Tra2b^fl/fl^; Nestin-Cre^tg/0^*) can be expected in a single litter (**Fig. 1A**). All four possible genotypes were equally distributed according to Mendelian law (**Fig. 1B**). Notably, all mice were born alive but KO mice died within the first hours of life. Only few KO mice survived up to a maximum of 36 hours, while HET mice had a normal life expectancy. Despite this drastic effect of *Tra2b* depletion, KO pups did not present major alterations in outer appearance (**Fig. 1C**). Previously we have shown that ubiquitous knock-out of *Tra2b* leads to early embryonic lethality in mice [21]. Here we demonstrate that the sole ablation of *Tra2b* in parts of the central nervous system causes perinatal lethality in mice and suggest that *Tra2b* is essential for neuronal development.

### Neuronal-specific *Tra2b* Knock-out Mice Show Severe Developmental Defects in the Brain and Aberrant Cortical Patterning

To understand the underlying cause of perinatal lethality we first histologically analyzed the brains of KO mice at various developmental stages using hematoxylin and eosin staining. Compared to control mice KO mice showed a dilation of both the third and lateral ventricles (ventriculomegaly) (**Fig. 1D**). The dilation of the ventricles had its onset at around 14.5 dpc and progressed until birth. Dilation at 14.5 dpc was still moderate and cortical patterning was comparable to control animals. At 16.5 dpc the lateral ventricles occupied a big part of the brain hemispheres and the cortical layers were no longer distinguishable. The ependyma which constitutes the ventricular lining appeared to be fully absent or destroyed in KO animals, even though the choroid plexus was present but being detached as a consequence of ventriculomegaly. Furthermore, late stage brain morphology (18.5 dpc) showed irregularities in the caudal regions of the septum or the anterior thalamic area. Heterozygous knock-out animals retained normal cortical patterning with normal ventricle sizes at all stages of development. This is in line with our finding of *Tra2b* expression being comparable to controls on brain sections (**Fig. 1E** **, Fig. S1**) and on Western Blots [17]. This suggests that a single gene copy of *Tra2b* can fully compensate protein function without producing an obvious phenotypic effect. The ventricular and subventricular zones of the developing mouse brain showed notably high *Tra2b* expression (**Fig. 1E**). However, *Tra2b* expression was strongly reduced in the ventricular and subventricular zones of KO animals. In general, brains of KO animals showed reduced levels of *Tra2b* expression but clearly retained localized centers of expression that likely comprise Nestin-negative cells (**Fig. 1E** **, Fig. S1**). This was in so far expected as the mosaicism of *Cre* expression is based on the natural expression pattern of the *Nestin* promoter. The absence or decay of the ventricular cortex layers in KO animals (**Fig. 1D** **, Fig. S1**) and the fact that these layers are loci of neuronal differentiation and neuronal outgrowth leading to cortex formation [56], [57] suggest that *Tra2b* plays a pivotal role in cortical development.

### Brain Malformations are Initiated by Massive Apoptosis in the Cortex Followed by the Loss of Proliferative Potential in Neurogenic Areas

**Figure 2:**
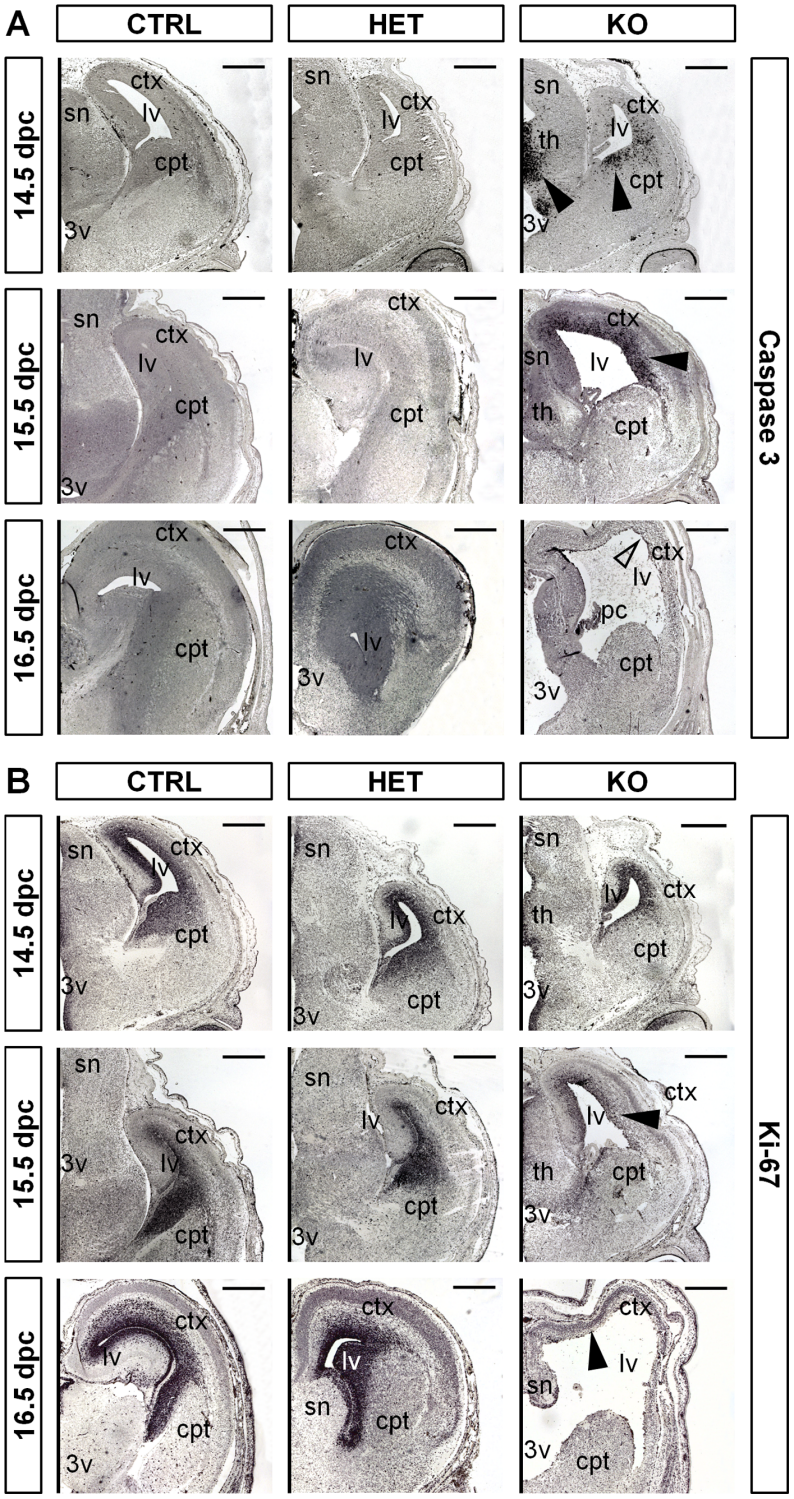
Brain malformations are initiated by massive apoptosis in the cortex. (**A**) Immunostaining for Caspase-3 on paraffin-embedded coronal sections indicates prominent apoptosis in the proximal cortical layers and in the thalamic area of 14.5 dpc and 15.5 dpc KO embryos (black arrowheads). Remaining cortical tissue does not show apoptosis at 16.5 dpc and later stages (light arrowhead). (**B**) Immunostaining for Ki-67 shows initial decrease of proliferation at 14.5 dpc which is fully lost at 16.5 dpc in KO animals (black arrowheads). Control and HET animals retain strong Ki-67 signals in the proximal cortical layers at all indicated developmental stages. Scale bar equals 400 µm; ctx, cortex; th, thalamus; cpt, caudoputamen; sn, septal nuclei; 3v, third ventricle; lv, lateral ventricle.

To further elucidate the course of the pathological progression we analyzed apoptotic events and the proliferative potential in KO versus control brains using immunostainings for cleaved caspase 3 (*Casp3*) and Ki-67. Localized presence of *Casp3* indicating apoptotic events is a typical hallmark of neuronal development as each proliferation, differentiation and apoptosis orchestrate neuronal restructuring [58], [59]. KO brains showed drastically elevated levels of Casp3 indicative of apoptosis at 14.5 and 15.5 dpc but not at later stages of development (**Fig. 2A**). This primarily affected the cortical layers close to the lateral ventricles and the anterior thalamic region between the cavities of the third ventricle. Accordingly, the thalamic area presented morphological anomalies at later stages of development (16.5 dpc). The more dorsally located septal nuclei appeared largely unaffected, however (**Fig. 2A, B**, KO 16.5 dpc).

In concordance with these apoptotic events the expression of the proliferation marker Ki67 started to decrease at around 14.5 dpc in cells of the ventricular and subventricular zone and was fully lost at 16.5 dpc and all later stages (**Fig. 2B**). This temporal occurrence of events suggests proliferation loss being a direct consequence of initial apoptotic events ultimately leading to tissue loss and ventriculomegaly. At present we cannot discern whether cells affected by apoptosis are neuronal progenitor cells or differentiated neurons. However, cells are affected most in close proximity to the ventricle (**Fig. 2A**). In a GFP-reporter mouse line *Nestin* has been shown to be expressed in precisely these regions, the ventricular and subventricular zone of the cortex [50]. This might indicate that affected cells are mostly neuronal progenitors. The brain development of HET animals was not affected. In these brains we neither detected an increase of apoptosis, nor a loss of proliferative potential.

This demonstrates that haplosufficiency of *Tra2b* has no phenotypic consequence and rules out any secondary effect of the *Nestin-Cre* transgene on brain development. In contrast, depletion of *Tra2b* in neuronal precursors leads to ventriculomegaly and aberrant cortical patterning. This appears to be a direct result of massive apoptotic events in ventricular and subventricular cortical layers and subsequent loss of the proliferative potential in these neurogenic areas. These findings clearly point out a pivotal role of *Tra2b* in neuronal development and survival.

### *Tra2b* Knock-down Efficiency is Variable between Individuals

**Figure 3:**
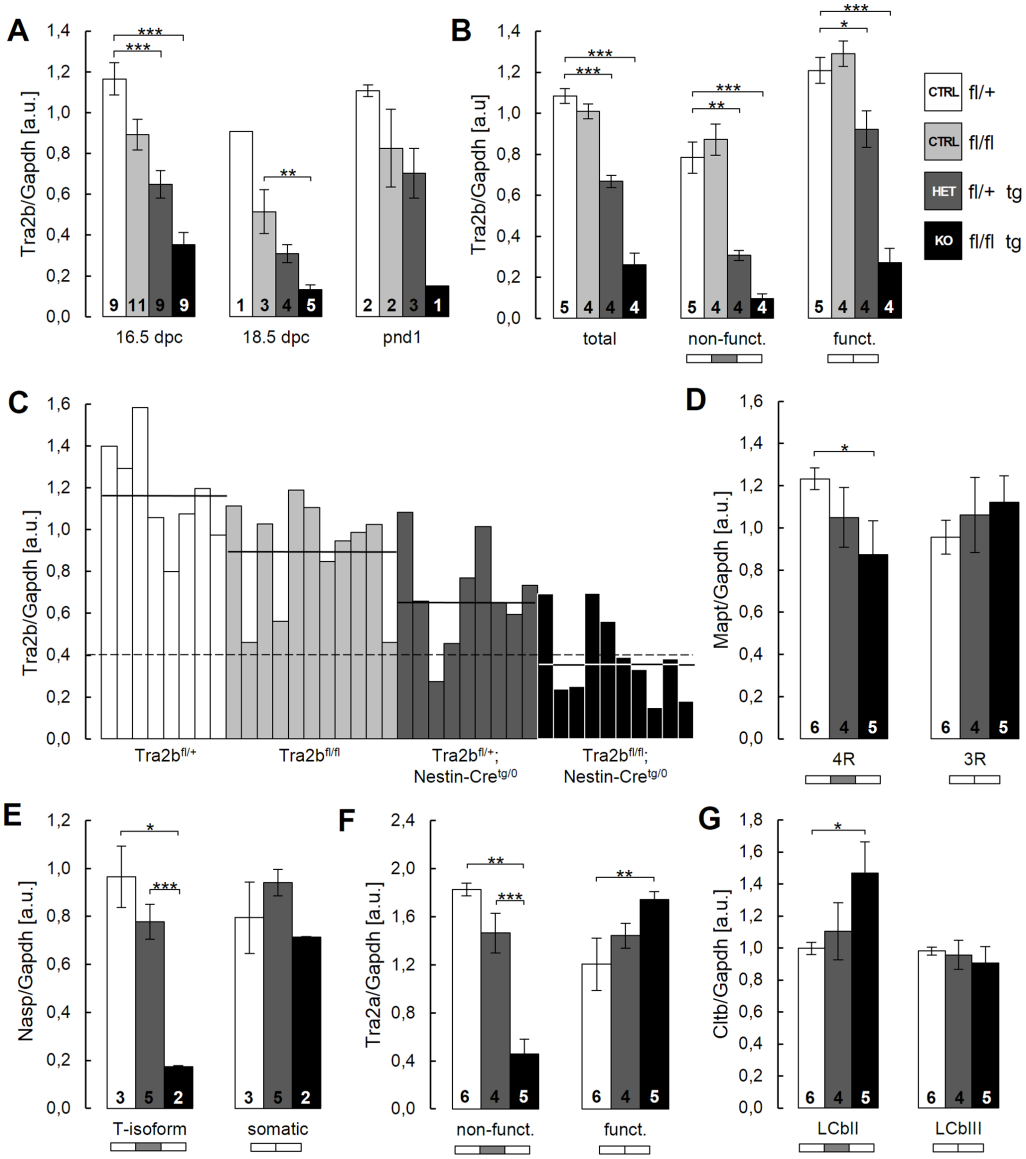
Bona fide *Tra2b*-related splicing events can be detected *in vivo* in conditional *Tra2b* knock-out mice. (**A–G**) Quantitative real-time PCR using whole brain RNA of control, HET and KO embryos at 16.5 dpc. All mRNA levels have been normalized to *Gapdh*. (**A**) Total *Tra2b* expression levels are effectively reduced in KO animals at 16.5 dpc, 18.5 dpc and PND1. (**B**) Expression levels of both the functional and non-functional *Tra2b* isoforms are reduced at 16.5 dpc. (**C**) Individual expression levels of total *Tra2b* largely behave accordingly to the respective genotype but are subject of variation within each group. Horizontal lines indicate the mean of each genotype group. The dashed line indicates a threshold value of 0.4. (**D**) Expression levels of the 3R and 4R isoforms of the *Mapt* transcript. Transcript levels of the 4R isoform are reduced in KO brains. (**E**) Expression levels of the LCbII and LCbIII isoforms of the *Cltb* transcript. Transcript levels of the LCbII isoform are reduced in KO brains. (**F**) Expression levels of the somatic and the testis-related isoform of the *Nasp* transcript. Transcript levels of the testis-specific T-isoform are drastically reduced by −5.5-fold in KO brains. (**G**) Expression levels of the functional and non-functional isoform of the *Tra2a* transcript. Transcript levels of the non-functional isoform are drastically reduced by −4.0-fold and levels of the functional isoform are increased by +1.4-fold. *Mapt*, microtubule associated protein tau; *Cltb*, Clathrin light chain b; *Nasp*, Nuclear autoantigenic sperm protein; *Tra2a*, Transformer 2 alpha; a.u., arbitrary units; error bars indicate the s.e.m.; significance levels are *p<0.05, **p<0.01, ***p<0.001 (Student’s t-test); N is given by numbers within bars.

Though all neuronal-specific KO mice presented ventriculomegaly, disturbed cortical patterning and perinatal lethality, the severity of the brain phenotype was variable between animals. Using whole brain RNA we first analyzed expression levels of *Tra2b* at different developmental stages. By real-time RT-PCR we found that *Tra2b* was effectively down-regulated in brains of 16.5 dpc, 18.5 dpc and PND 1 KO animals (**Fig. 3A**). As whole brain RNA has been used, residual *Tra2b* expression is likely a result of *Nestin*-negative cells that do not express *Cre* recombinase. As expected, HET mice showed a moderate but significant down-regulation of *Tra2b* transcripts. In control animals the total expression of *Tra2b* in the brain was largely constant between 16.5 dpc and birth (**Fig. 3A**).

*Tra2b* is known to autoregulate its expression level via a splicing feedback-loop by including cassette exon 2 into the transcript [60]. Exon 2 contains a premature termination codon rendering the transcript non-functional and thus decreasing the Tra2b protein concentration. Using isoform-specific real-time PCRs we found a significant down-regulation of both the functional (−78%) and non-functional (−88%) *Tra2b* isoforms (**Fig. 3B**). In general the down-regulation of the non-functional isoform was stronger than for the functional isoform. Interestingly, in HET mice the levels of functional transcripts were only mildly reduced (−24%) while non-functional transcripts were very strongly reduced (−61%). These data suggest that the auto-regulatory splicing feedback-loop is functional in mice [17]. The only mildly altered levels of functional *Tra2b* transcripts are consistent with survival and normal brain development in HET mice. This suggests that loss of a *Tra2b* gene copy can be compensated to a great extent via this auto-regulatory mechanism.

Though average *Tra2b* knock-out was effective in KO mice we found individual differences between single animals while comparing animals of all genotypes at 16.5 dpc (**Fig. 3C**). Despite transcript levels were on average gradually reduced according to the genotype (horizontal lines), we found single KO animals with *Tra2b* transcripts hardly lower than in control animals. Variability and mosaicism in *Cre* expression throughout the brain of different individuals might explain that effect. To still allow functional analysis of splicing events in *Tra2b* knock-out mice we chose KO mice with relative *Tra2b*/*Gapdh* transcript levels of lower than 0.4 (**Fig. 3C**, dashed line).

### Physiological Splicing Events of Bona Fide Tra2b Targets can be Reconstituted in Neuronal-specific *Tra2b* Knock-out Mice

To check whether relative *Tra2b*/*Gapdh* levels lower than 0.4 have an impact on physiological splicing events in the brain we quantified the isoform splicing of bona fide *Tra2b* target transcripts. Using isoform-specific real-time PCRs we analyzed the splicing of the well-known splicing targets microtubule associated protein tau (*Mapt*) exon 10 [25], [26], [27], the vesicle coating protein clathrin light chain b (*Cltb*) exon 5 [15], [29], the nuclear autoantigenic sperm protein (*Nasp*) exon 7 and the *Tra2b* paralog transformer 2 alpha (*Tra2a*) exon 2 [17], [61]. For *Mapt* (**Fig. 3D**), *Nasp* (**Fig. 3E**) and *Tra2a* (**Fig. 3F**) inclusion of the respective cassette exon into the transcript is promoted by Tra2b. Accordingly, we found a significant decrease in the *Mapt* 4R, *Nasp*-T and non-functional *Tra2a* isoforms. This demonstrates that these Tra2b-dependent exons are more frequently skipped in KO mice. For the *Cltb* transcript Tra2b promotes skipping of exon 5 [15], [29]. Accordingly, we found a significant increase in the exon 5 containing LCbII isoform in KO mice (**Fig. 3G**). In summary, these data demonstrate that our neuronal-specific *Tra2b* knock-out system can reconstitute well-known splicing events in the developing murine brain. Consequentially, we sought to perform an exome-wide screening for alternative splicing events in the developing central nervous system using whole exome array technology.

### Identification of Tra2b Targets via Mouse Exon Array Analysis

**Figure 4:**
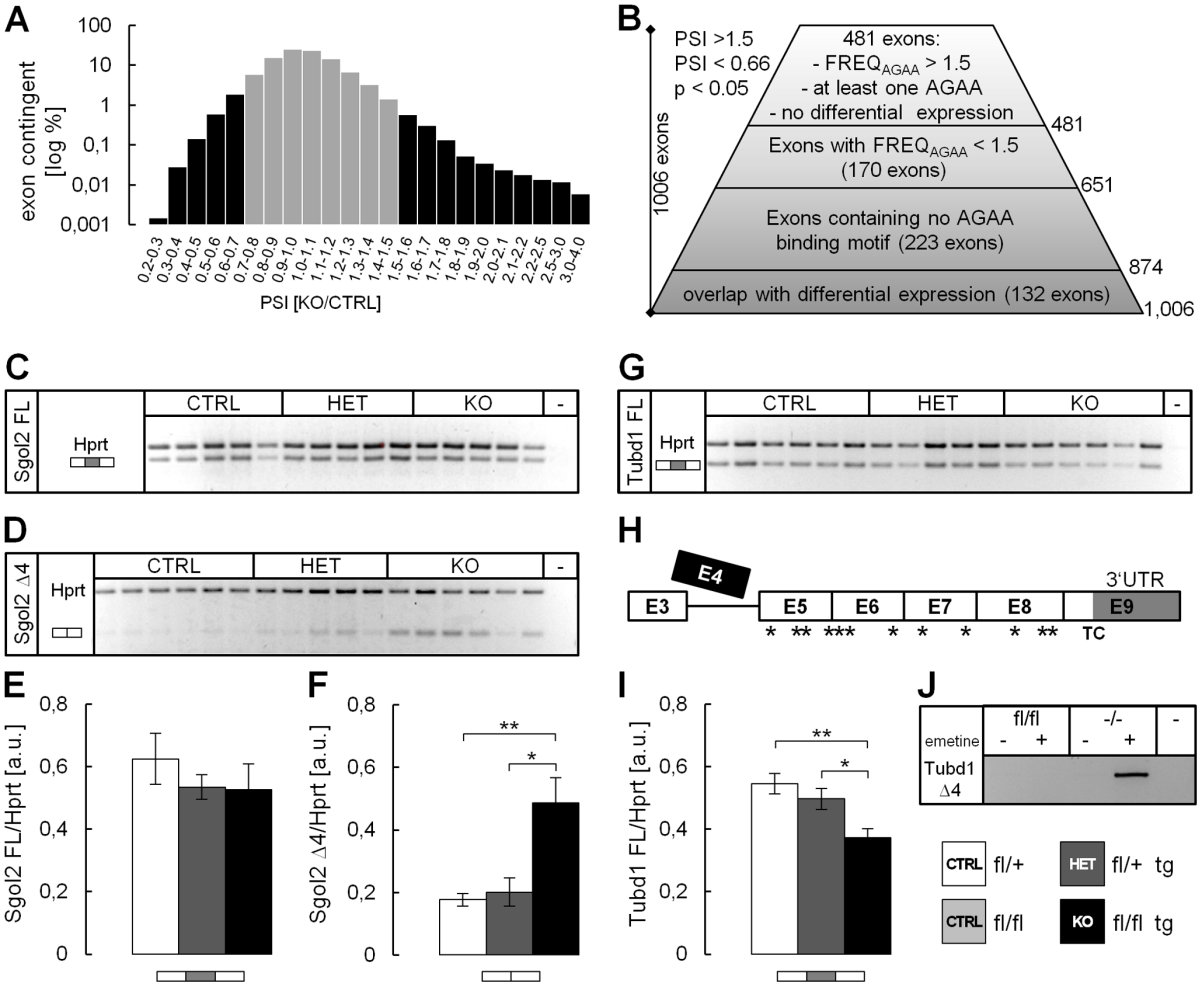
Mouse whole exon array analysis reveals *Tubd1* exon4 and *Sgol2* exon4 as *in vivo* targets of *Tra2b*. Whole brain RNA of 4 CTRL animals and 4 KO animals was analyzed on mouse exon array. (**A**) The inclusion ratio (PSI, percent splicing inclusion) of each identified exon is defined as [PSI_KO]/[PSI_CTRL]. PSI distribution reached from ∼0.2 until ∼4.0. (**B**) Initial filtering strategies comprised exclusion of PSIs between 0.66 and 1.5 (grey bars) as well as restriction to p-values smaller than 0.05 which yielded a total of 1,006 exons. Exons associated with transcripts identified as being transcriptionally up- or downregulated were excluded from analysis. Ranking of those was further refined using large PSI values and considering presence of putative Tra2b binding sites (AGAA-motifs). Thereby, exons had to contain at least a single AGAA-site and a AGAA-frequency higher than 1.5. (**C,D,G,H**) Semi-quantitative RT-PCT on whole brain RNA was carried out using isoform specific primers for *Sgol2* FL (**C**), *Sgol2* Δ4 (**D**), *Tubd1* FL (**G**) and *Tubd1* Δ4 (**H**) confirming splicing events identified on the microarray. All isoform expression levels were densitometrically measured and normalized against *Hprt* (**E,F,I**). The *Tubd1* Δ4 isoform could not be detected using whole brain RNA, as skipping of exon4 introduces numerous premature termination codons leading to nonsense-mediated decay of the transcript (**H**). Treatment of wt and *Tra2b*-depleted murine embryonic fibroblasts with emetine successfully inhibited NMD and the *Tubd1* Δ4 isoform was detectable in *Tra2b*-depleted cells only (**J**). FL, full length; Δ4, transcript lacking exon 4, (−) PCR negative control; a. u., arbitrary units; error bars show the s.e.m.; significance levels are *p<0.05, **p<0.01, ***p<0.001 (Student’s t-test).

To identify novel splicing targets for Tra2b we performed exome analyses of four CTRL and four KO whole brain RNAs from 16.5 dpc pups using Affymetrix mouse exon 1.0 ST arrays. By quantifying total *Tra2b* levels we assured a strong *Tra2b* knock-down with normalized expression levels of lower than 0.4 in the analyzed samples (see **Fig. 3C**). From the mean values of the CTRL and KO group we calculated global changes in the expression of whole transcripts and exon-specific differences, which would point out differences in splicing inclusion (PSI, percentage of splicing inclusion). Most of the identified exons showed no or very little changes in splicing inclusion (**Fig. 4A** **,** gray bars). As a threshold for PSI changes we filtered out exons with less than ±1.5-fold up- or downregulation, which excludes about 97% of the identified exons. Furthermore, exons with significance levels of worse than 0.05 were filtered out, still yielding a list of 1,006 differentially included exons (**Fig. 4B** **, Dataset S1**). Unexpectedly, we found multiple exons from one and the same gene being identified as alternatively spliced (132 exons in total). We compared the list of 86 differentially expressed transcripts (**Dataset S2**) with those genes called with multiple exons and found that the 132 alternatively spliced exons were matching with 54 out of 86 differentially expressed genes. We analyzed a subset of double-identified transcripts including the transcription factors *Eomes*, *Tcf4* and others and found general changes in expression but no exon-specific changes (**Fig. S2**). This indicates that these exons being identified to be alternatively spliced are truly artifacts caused by misinterpretation of altered transcript levels. Thus, exons belonging to this group were excluded from our candidate list (**Fig. 4B**). The generally very high number of alternative events and the strong overlap with differentially expressed transcripts is probably indicative of poor data quality and a high error rate which strongly increases the number of false positive exons. We propose that the strongly altered brain morphology and misdevelopment in the KO mice and the according changes in cell type and transcriptome composition cause a large number of artifact results. Furthermore, mosaicism of *Cre* recombinase expression leads to two distinct cell populations in the KO brain. First, surviving cells in which *Tra2b* was depleted and show altered splicing patterns and, second, cells that express *Tra2b* and show normal splicing. In such a mosaic brain the latter cell population masks the splicing effect present in KO cells. That phenomenon appears to hamper our efforts to reliably call alternative splicing events.

As an attempt to counteract this problem we excluded exons that do not contain any AGAA binding motif (**Fig. 4B**). Considering that such motif occurs statistically once in a 256 bp stretch we calculated the binding site frequency (F) as the number of binding sites multiplied with the exon length in bp divided by 256. Exons with F <1.5 were excluded resulting in 481 remaining exons (**Fig. 4B** **, Dataset S3**). To verify candidate exons we performed isoform-specific semi-quantitative RT-PCRs for a subset of 20 selected candidates (**Fig. S2, and data not shown**). We were largely unable to validate splicing events of candidate exons as the expression of both respective isoforms was either indifferent between CTRL and KO animals (e.g. *Slu7*, *Nrg1*) or one of the two isoforms was not detectable in the brain at all (e.g. *Sox6*) (**Fig. S2C**). However, splicing events of the Shugoshin-like2 and Tubulin delta chain 1 transcripts could be validated in our candidate screening (data not shown). Therefore, both transcripts were selected for further analysis via RT-PCR and minigene splicing assay. Results obtained from microarray analyses imply that the majority of data output from the mouse exome array are false positive artifacts based on either general transcript loss due to cell loss and misdevelopment or drastic changes in tissue and cell-type distribution in the severely affected KO brains. We propose employing a more homogeneous knock-out cell system is necessary for a successful exome-wide screening approach – especially to reliably detect changes in exon splicing.

### Shugoshin-like 2 (*Sgol2*) Exon 4 and Tubulin Delta Chain 1 (*Tubd1*) Exon 4 are *in vivo* Targets of Tra2b

Using isoform-specific semi-quantitative RT-PCR we were able to reproduce the splicing pattern of Shugoshin-like 2 (*Sgol2*) and Tubulin delta chain 1 (*Tubd1*). While the isoform of *Sgol2* including exon 4 only showed a modest and non-significant decrease between CTRL and KO brains, the Δ4 isoform significantly increased by 2.75-fold thereby resembling the 2.7-fold loss of exon inclusion detected on the microarray (**Fig. 4** **C–F, Dataset S3**). These data demonstrate that the inclusion of exon 4 into the *Sgol2* transcript is promoted by Tra2b. Similarly, we detected a splicing response for exon 4 of the *Tubd1* transcript. The isoform including exon 4 showed a significant decrease by 1.46-fold in the brains of KO animals compared to CTRL (**Fig. 4G–J**) resembling the 1.7-fold loss of exon inclusion detected on the microarray (**Dataset S3**). Surprisingly, we were unable to detect the Δ4 isoform of *Tubd1* in brains of either CTRL or KO mice. Computational analysis showed that skipping of exon 4 from the *Tubd1* transcript results in at least 10 premature termination codons that would likely lead to transcript degradation by nonsense-mediated decay (**Fig. 4H**). To test this hypothesis we used emetine to block NMD in *Tra2b*-depleted and *Tra2b*-expressing murine embryonic fibroblasts. In *Tra2b*-depleted cells treated with emetine the Δ4 isoform of *Tubd1* was strongly detectable while it was not detectable in *Tra2b*-expressing cells (**Fig. 4J**). This strongly suggests that skipping of *Tubd1* exon4 results in nonsense-mediated decay of the transcript and implicates splicing regulation of that exon to be a possible mechanism of expressional regulation *in vivo*.

### Alternatively Spliced Exons of *Sgol2* and *Tubd1* are Responsive to Depletion and Overexpression of *HTRA2B* in a Minigene Splicing Assay

**Figure 5:**
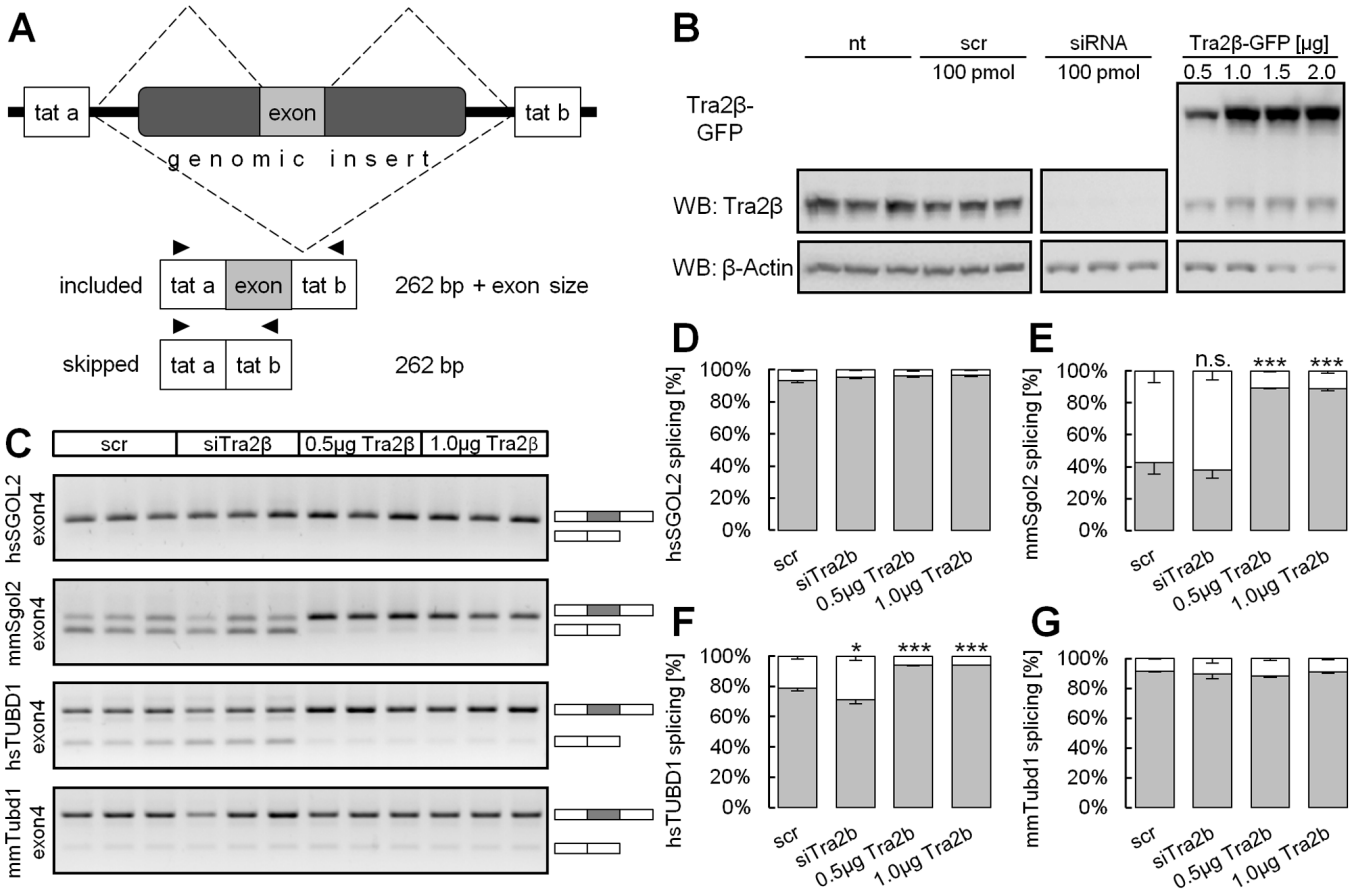
Splicing of *Sgol2* and *Tubd1* is responsive to changes in Tra2b concentration. (**A**) The murine and human versions of the genomic regions comprising the identified exons of *Sgol2* and *Tubd1* were cloned into the pSPL3 exon trapping vector. (**B**) HEK293T cells were co-transfected with the pSPL3 minigene vector and siRNA specific for *Tra2b* or a *TRA2B-GFP* expression vector. Western Blot analysis shows efficiently reduced Tra2b protein levels and solid overexpression of TRA2B-GFP. (**C**) RNA was analyzed for exon inclusion after 48 h by semi-quantitative RT-PCR. (**D–G**) RT-PCR results were densitometrically quantified. Exon 4 of murine but not human *Sgol2* is responsive to increased concentrations of Tra2b as splicing inclusion significantly increased from 43% to 89%. Knock-down of *Tra2b* is insufficient to reduce *Sgol2* exon4 splicing inclusion. Exon 4 of human but not murine *Tubd1* is responsive to increased concentrations of Tra2b as splicing inclusion significantly increased from 79% to 94%. Knock-down of *Tra2b* decreased inclusion of exon 4 from 79% to 71%. Nt, non-treated; scr, scrambled siRNA; si, siRNA against *Tra2b*; n.s., non significant; error bars show the s.e.m.; significance levels are *p<0.05, **p<0.01, ***p<0.001 (Student’s t-test).

We further tested splicing of the *Sgol2* and *Tubd1* alternative exons in a minigene splicing assay. Exons with at least 0.5 kb of flanking intronic sequence were cloned into the pSPL3 vector (**Fig. 5A**). Transfected into HEK293T cells the vector produces mRNA containing the genomic insert with the respective exon flaked by the *tat a* and *tat b* exons of HIV. These flanking exons are constitutively spliced resulting in a specific mRNA. Inclusion of the exon of interest results accordingly in a longer mRNA that can be detected and compared by size differences using RT-PCR (**Fig. 5A**). We tested the splicing of the human and murine versions of each exon while overexpressing or depleting *HTRA2B* using siRNAs (**Fig. 5B**). PCR results indicate that only the murine *Sgol2* but not the human *SGOL2* is responsive to *HTRA2B* overexpression in HEK293T cells (**Fig. 5C–E**). Under normal conditions (scr) *Sgol2* exon 4 was included in 43% of the transcripts. After overexpression of *HTRA2B* exon 4 was included in 89% of the transcripts suggesting that *HTRA2B* effectively promotes inclusion of murine *Sgol2* exon 4 (**Fig. 5E**). On the contrary, *HTRA2B* overexpression had no effect on the human *SGOL2* transcripts (**Fig. 5C**). The human exon 4 is included in 93% of the transcripts under normal conditions (scr) (**Fig. 5D**). This suggests that *HTRA2B* overexpression is unable to further enhance *SGOL2* exon 4 inclusion. As we used human embryonic kidney cells (HEK293T) for minigene splicing assays we analyzed the intrinsic splice site strengths of the tested exons by comparing to the primate splice site consensus (http://ibis.tau.ac.il/ssat/SpliceSiteFrame.htm). Indeed, scores of both the 3′ and 5′ splice sites were higher for the human sequence compared to the murine version. Furthermore, ablation of endogenous *HTRA2B* in HEK293T cells by RNAi did not decrease exon 4 inclusion (**Fig. 5** **D, E**). These results suggest that the high intrinsic splice site strength of the human *SGOL2* exon 4 is sufficient to constitutively splice that exon. Therefore, results obtained for *Sgol2* in the mouse are likely not transferable to humans.

The alterantive splicing of tubulin delta chain exon 4 in the minigene is *HTRA2B* dose-dependent (**Fig. 5C**). Under normal conditions (scr) 79% of transcripts show exon 4 inclusion. Ablation of *HTRA2B* slightly but significantly reduced exon 4 inclusion to 71% while overexpression significantly increased inclusion to 94% (**Fig. 5F**). This indicates that *HTRA2B* promotes *TUBD1* exon 4 inclusion, confirming our results obtained in the mouse model. Surprisingly, the murine version of the minigene failed to respond to altered *HTRA2B* concentrations (**Fig. 5C**). Under normal conditions (scr) exon 4 was included in 92% of the transcripts (**Fig. 5G**) and splicing inclusion did not change upon knock-down or overexpression of *HTRA2B*. This finding is in so far intriguing as we originally detected the *Tubd1* exon 4 splicing event in mouse (**Fig. 4** **G, I**). The splice site strengths of human and murine *Tubd1* exon 4 are very similar and analysis suggest that the murine 3′ and 5′ splice sites are presumably equally well recognized by the human splicing machinery in HEK293T cells. However, there are minor differences in number and position of well-known binding sites (e.g. SF2/ASF and SRp40) that might be attributable to a more efficient inclusion of exon 4 in a human cellular system compared to native conditions in the mouse. Whether and to what extend splicing processes of *Sgol2* and *Tubd1* have a physiological impact *in vivo* remains to be clarified.

### Cyclin Dependent Kinase Inhibitor 1a (*p21*) is Upregulated in Neuronal Specific *Tra2b* Knock-out Mice

**Figure 6:**
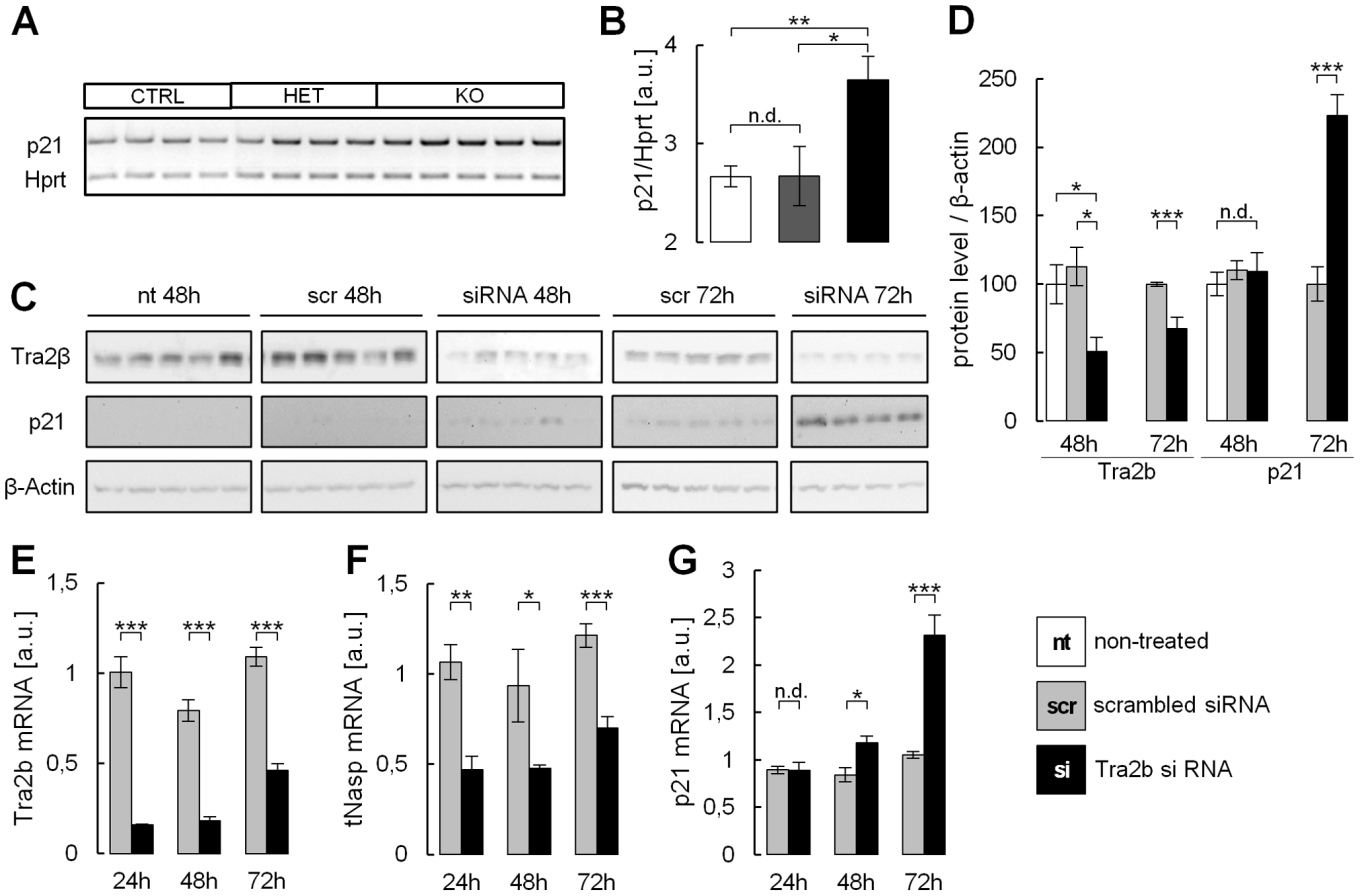
*p21* is upregulated as a response to *Tra2b* depletion in the mouse brain and in neural stem cells. (**A,B**) Semi-quantitative RT-PCR using whole brain RNA of neuronal specific *Tra2b* KO mice and controls. *p21* is significantly upregulated by 1.5-fold in KO mice as compared to HET or control mice. *p21* expression is indifferent between controls and HET mice. *p21* expression was normalized to *Hprt*. (**C–G**) NSC34 neural stem cells were transfected with siRNAs specific to *Tra2b* or scrambled siRNAs. siRNA treatment but not scr-treatment effectively reduced Tra2b protein and mRNA levels after 24 h, 48 h and 72 h after transfection (**C–E**). Tra2b function was strongly reduced as the *Nasp* transcript showed a significantly lower inclusion of the T-exon at 24 h, 48 h and 72 hours after transfection (**F**). 48 hours after transfection *p21* expression was found slightly but significantly increased on RNA level (**G**) but not on protein level (**D**). 72 hours after transfection p21 was massively and highly significantly upregulated on RNA and protein level by +2.2-fold (**D,G**). a.u., arbitrary units; nt, non-treated; scr, scrambled siRNA; si, siRNA against *Tra2b*; error bars show the s.e.m.; significance levels are *p<0.05, **p<0.01, ***p<0.001 (Student’s t-test).

In our previous study we identified the T-exon of the histone chaperone *Nasp* to be spliced by Tra2b in mouse testis and the developing brain [17]. Ablation of *Tra2b* decreases the inclusion rate of the *Nasp* T-exon resulting in a decreased expression of the *tNasp* isoform (**Fig. 3E**). Selective ablation of *tNasp* via RNAi has previously been demonstrated to induce apoptosis in tumor cells via expressional upregulation of the cyclin dependent kinase inhibitor 1a (*Cdkn1a/p21,* ENSMUSG00000023067) [35]. In agreement to this we found *p21* to be upregulated by more than 1.5-fold in KO mice in our exon array analysis (**Dataset S2**). Using semi-quantitative RT-PCR we validated these exon array-based data. mRNA levels of *p21* were significantly upregulated by 1.4-fold in a group of KO animals compared to controls or HET mice (**Fig. 6A, B**).

Due to *Cre* recombinase-related mosaicism in the brain of KO mice we expect that apoptotic cells will contribute only with a small fraction to the whole brain (**Fig. 2A**). Still we were able to detect *p21* upregulation in whole brain RNA indicating a solid upregulation in the affected cell populations. To test this hypothesis we used murine neuronal stem cells (NSC34 cells) as their neuronal fate resembles the neuronal identity of affected cells found in the ventricular and subventricular cortical layers of KO mice [54]. NSC34 cells were depleted of *Tra2b* using specific siRNAs. RNA levels of the functional *Tra2b* Δ2 isoform and Tra2b protein levels were markedly reduced within 24 and 48 hours after transfection (**Fig. 6C–E**). As expected depletion of *Tra2b* caused changes in splicing of the *Nasp* transcript. The *tNasp* isoform was significantly downregulated by at least 50% within 24 to 48 hours after transfection (**Fig. 6F**) suggesting that Tra2b activity is functionally abolished. To test whether *p21* elevation found in KO mice (**Fig. 6A, B**) can be reconstituted in NSC34 cells, we measured *p21* expression via quantitative real-time PCR and determined p21 protein amounts via Western Blot. Within 48 hours after transfection p21 did not increase on protein level (**Fig. 6C, D**). However, we found a modest but significant increase of *p21* expression on mRNA level (**Fig. 6G**). To asses long term effects of *Tra2b* ablation on *p21* expression we assayed cells 72 and 96 hours after transfection. Notably, after 72 hours about 20% of the *Tra2b*-depleted cells had died (data not shown) and *p21* was strongly and highly significantly upregulated by +2.2-fold on mRNA and protein level (**Fig. 6C, D, G**). Strikingly, after 96 hours the majority of transfected cells had died indicating a massive loss of proliferative potential and possible cell cycle arrest and apoptosis. The succession of events suggests that the downstream effects originally triggered by *Tra2b* ablation do not occur instantly but rather require time to establish. This is in line with our findings in KO mice, in which onset of *Cre* recombinase expression and thus *Tra2b* depletion starts at around 10.5 dpc. Abnormalities in brain development cannot be observed before 14.5 to 15.5 dpc, however. These findings indicate that *Tra2b* deficiency leads to *p21* activation and suggest *p21* activation to be a major contributor to apoptosis and developmental defects observed in neuronal-specific *Tra2b* KO mice.

## Discussion

Alternative splicing is a complex and extremely potent mechanism to modulate gene function and individual splicing factors can exert an effect on a specified group of transcripts [1], [45]. Many features of the general splicing machinery are well understood but little is known about the function and target transcripts of non-essential splicing factors. Mice deficient for the splicing activator *Tra2b* are embryonically lethal at early gestational stages [21]. Here we generated a neuronal-specific knock-out mouse model for *Tra2b*. These mice are born but die shortly after delivery and display severe developmental defects in the cortical and thalamic brain regions (**Fig. 1** **,** **2**). Degeneration of these regions is preceded by massive apoptotic events resulting in a loss of proliferation. Here we demonstrate that the cyclin dependent kinase inhibitor 1a (*p21*) is drastically upregulated in the affected brains and in *Tra2b*-depleted neuronal progenitor cells (**Fig. 6**). These data indicate that Tra2b is essential for the survival of neuronal precursors and plays a pivotal role in neuronal development.

Immunohistological analyses of the developing mouse brain showed strongest expression of Tra2b in ventricular cell layers of the cortex and the thalamus (**Fig. 1E**). Neuronal-specific KO mice show severe morphological alterations in these brain regions. Massive apoptosis in the ventricular and subventricular zone of the cortex and in the thalamus are followed by a drastic reduction in expression of the proliferation marker *Ki67*. The ventricular zone is a neurogenic area and origin of neuronal migration to form the layered structure of the cortex [62]. Consequently, cell death and the incapacity to proliferate are detrimental for normal cortex formation.

HET animals did not show any alterations in brain development (**Fig. 1** **,** **2**), had normal life expectancy and did not demonstrate behavioral abnormalities. This suggests that a single gene copy is sufficient to provide the required Tra2b protein levels. Concordantly, in HET mice the reduction of the functional *Tra2b* isoform lacking exon 2 was smaller than the reduction of the non-functional isoform (**Fig. 3B**). This suggests that the *Tra2b* auto-splicing mechanism [60] is functional in the murine brain. This largely compensates the loss of a single gene copy as Tra2b protein levels in HET mice are virtually indistinguishable from controls [17]. Furthermore, the normal brain development of heterozygous animals (i.e. *Tra2b^fl/+^; Nestin-Cre^tg/0^*) excludes any genotoxic stress emerging form high levels of *Cre-recombinase* expression, which has been shown to cause hydrocephaly and reduction of cortical cell proliferation in mice [63].

A less severe cortical phenotype has recently been demonstrated in a conditional *Emx1-Cre Tra2b* mouse model [64]. These mice were fully viable and did not show gross behavioral abnormalities, suggesting that *Tra2b* cortical loss can be tolerated. In contrast, depletion of *Tra2b* by *Nestin-Cre* in neuronal progenitor cells affects a broader range of neuronal cells, which showed to be essential for survival. Indeed, by using a reporter line for this Nestin-Cre mouse, strong expression throughout the central and peripheral nervous system has been demonstrated [48]. Due to mosaic effects morphological changes of the brain, total expression levels of *Tra2b* and detectable effects on Tra2b splicing targets were variable between animals of the same genotype (**Fig. 3C**). To circumvent this problem we excluded animals with insufficient *Tra2b* knock-down (*Tra2b*/*Gapdh* ratio >0.4) from our analyses. This allowed solid *in vivo* validation of bona fide Tra2b target transcripts in the mouse brain (**Fig. 3D–G**) turning these neuronal-specific mice into a suitable model for the identification of novel physiological *Tra2b* splicing targets.

In the frame of this study we performed mouse exon array as well as whole transcriptome sequencing analysis (data not shown) to identify novel Tra2b splicing targets. Neither of these two approaches was able to reliably call splicing targets of Tra2b. Similar to exon array analysis also whole transcriptome sequencing yielded numerous top ranking candidates that we were unable to validate by RT- and qRT-PCR. We propose that such high throughput screening approaches require a strictly homogeneous cellular system to gather starting material (RNA) from. In this study whole brain RNA of knock-out and control brains was used. We propose that the cellular composition of KO brains is too severely altered due to gross morphological changes, which does not allow a reliable comparison of relative exon abundances between KO and control brains. Likely, collection of RNA from a homogeneous cell culture or from material obtained by laser dissection would provide more accurate data.

By filtering and enrichment of exons containing Tra2b binding sites we identified *Sgol2* exon 4 as a Tra2b splicing target (**Fig. 4C–F**). Shugoshin protects the integrity of the cohesin-complex which regulates sister chromatid cohesion during meiosis I [65], [66]. It has been demonstrated to be essential for meiosis but not for mitosis and *Sgol2*-deficient mice are viable but infertile. Whether skipping of exon 4 from the *Sgol2* transcript has any impact on protein function or stability remains to be determined. Physiological implications of this splicing process in brain development are unclear. Moreover, we identified *Tubd1* as a Tra2b splicing target (**Fig. 4G–J**). Delta tubulin is highly expressed in testis and is a component of the perinuclear ring of the manchette, which helps to translocate and elongate the nucleus during sperm cell maturation [67], [68]. The somatic function of delta tubulin is poorly understood and expression levels in brain are low. Thus, the physiological relevance to brain development remains elusive. Skipping of exon 4 from the *delta tubulin* transcript causes a frameshift resulting in numerous premature termination codons. Our results indicate that skipping of exon 4 results in NMD (**Fig. 4H, J**). We propose that Tra2b-mediated splicing of *Tubd1* exon 4 is a mechanism to regulate *Tubd1* expression on a post-transcriptional level. During spermatogenesis Tubd1 is part of the perinuclear ring which forms during the transition from round to elongating spermatids [67]. This transition is preceded by massive *Tra2b* expression during spermatocyte and round spermatid stages [17]. We speculate that elevated *Tra2b* levels in round spermatids might increase *Tubd1* expression via exon 4 inclusion to facilitate transition into spermatid elongation.

Here we show that previously identified splicing targets of Tra2b show altered splicing patterns in *Tra2b*-deficient brains. These targets include Clathrin light chain b (*Cltb*), Microtubule associated protein tau (*Mapt*), the *Tra2b* paralog *Tra2a* and the Nuclear autoantigenic sperm protein (*Nasp*). We cannot exclude with absolute certainty, that splicing changes of these transcripts were detected due to gross morphological changes of the brain, which causes major alterations in cell type composition. The loss of specific cell types could directly cause downregulation of specific isoforms. However, all validated exons showed the expected mode of splicing with regard to splicing inclusion or skipping by Tra2b. Furthermore, the neuronal-specific isoform of *Cltb* (exon 5 included) was shown to be upregulated in KO mice, despite apoptosis and loss of neurons. We speculate that the real de-repression of *Cltb* exon 5 inclusion in KO mice is truly stronger than actually determined, as upon death of Tra2b-negative cells, Tra2b-expressing cells will dominate and mask the actual splicing changes.

Whether and to what extent missplicing of the above mentioned transcripts contributes to aberrant brain development in neuronal-specific KO mice cannot be defined with certainty. However, there are strong implications as some targets are closely related to neuronal function and disease.

Clathrins are functionally versatile proteins mainly involved in vesicle coating and regulation of endocytosis (reviewed in [69]). Splicing of exon 5 (also exon EN) of *Cltb* was shown to be indirectly regulated by Tra2b in a negative fashion *in vitro* [15]. Here we report that this repressive mode of splicing modulation by Tra2b is physiologically detectable in the murine brain (**Fig. 3G**). The neuronal *Cltb* isoform II (exon 5 included) comprises regulatory features for the C-terminal calmodulin binding domain. This isoform has been demonstrated to be sensitive to calcium and it has been proposed that skipping of *Cltb* exon 5 (isoform III) might keep vesicles in a calcium-insensitive state in non-neuronal tissue [29], [30]. In neurons this might represent a calcium dependent regulatory feature for vesicle release and recycling [70], [71], [72]. Altered splicing of *Cltb* exon 5 might therefore perturb the responsiveness of synaptic vesicles to calcium and cause impact synapse formation and function.

The microtubule associated protein Tau (*Mapt*) regulates microtubule stability and assembly and is associated with maintenance of neuronal morphology (reviewed in [73]). Tau and especially missplicing of exon 10 which encodes 1 out of 4 microtubule binding domains is involved in numerous neurological disorders and has been studied extensively [26], [27], [74], [75]. Under normal conditions the ratio between the 3R (exon 10 skipped) and the 4R isoform (exon 10 included) is tightly regulated by Tra2b-dependent splicing processes. This defined ratio is a prerequisite for normal microtubule function and stability and has been shown to be shifted by increased exon 10 usage in postmortem Alzheimer’s disease brains [25]. Here we confirm *Tra2b*-related changes in *Mapt* exon 10 usage in the developing mouse brain (**Fig. 3D**). Thus, Tau isoform ratios are perturbed in KO animals and might therefore elicit neurodegenerative processes and contribute to the observed brain phenotype.

The nuclear autoantigenic sperm protein (*Nasp*) is a histone chaperone that is expressed as two distinct isoforms: The *sNasp* (somatic) isoform and the *tNasp* (testis or embryonic) isoform that we have previously demonstrated to be expressed in testis and embryonic brain [17] (**Fig. 3E**). Splicing regulation of the *tNasp*-specific cassette-exon is facilitated by numerous Tra2b binding sites which are evolutionarily highly conserved. The tNasp protein has previously been demonstrated to affect progression of the cell cycle, as its absence causes failure in passing the G_1_/S-phase border [34]. Furthermore, *tNasp* ablation did lead to apoptosis of prostate cancer cells via an upregulation of *p21* (*Cdkn1a*) [35]. *P21* is a well known inhibitor of the cell cycle via inhibition of the cyclin-dependent kinases CDK2 and CDK4, which leads to G_1_-phase cell cycle arrest [76]. Strikingly, we detected an increase in *p21* expression in neuronal-specific *Tra2b* KO brains on mouse exon arrays, which we could confirm by RT-PCR (**Fig. 6A,B**).

Here we demonstrate for the first time (to our knowledge) a direct link between *tNasp* expression and cell cycle progression *in vivo* in the developing murine brain. Moreover, we validated our *in vivo* findings in a neuronal precursor cell system. NSC34 cells showed massive *p21* activation and died upon *Tra2b* knock-down which induced *tNasp* depletion (**Fig. 6C–G**). In line with aberrant brain development in neuronal-specific *Tra2b* KO mice, *p21* has been shown to restrict neuronal proliferation in the subgranular zone of the dentate gyrus of the hippocampus [77] giving an example of how *p21* expression can impact CNS development. Hence, *tNasp* ablation upon Tra2b-induced missplicing might strikingly contribute to cell death and the observed *p21* activation and apoptosis in neuronal-specific *Tra2b* KO mice, strongly accounting for impaired brain development. It cannot be excluded however, that onset of apoptosis is a result of increased intracranial pressure (normal pressure hydrocephalus) which can be caused by reduced resorption of cerebrospinal fluid or duct obstruction in the ventricular system [78].

Given the established role of tNasp as a histone chaperone or transporter [79], [80] it appears intriguing that sole ablation of *Histone H1* (independently of *Nasp*) causes cell cycle arrest and altered expression of cell cycle-related genes including *p21* [81]. We propose that *Tra2b*-dependent missplicing of *Nasp* results in deficient histone import to the nucleus and causes failure to pass the G_1_/S-phase checkpoint via p21 cell cycle inhibition.

Mice ubiquitously depleted of *Tra2b* die at early stages of gestation [21], which precludes detailed analyses of a systemic upregulation of *p21* during embryogenesis. A strong reduction in proliferative potential could well explain developmental arrest. Interestingly, mice ubiquitously depleted of *tNasp* die evenly early during embryonic development [34]. It will be of particular interest to show in future research whether high amounts of the histone importer *tNasp* are generally necessary to maintain quickly proliferating embryonic or tumor cells viable. *Tra2b* gained increasing significance in cancer research as its expression has shown to be elevated in lung, ovarian and cervical carcinomas (reviewed in [82]). Thus, inhibition of *Tra2b* or *tNasp* could probably be exploited to slow down or to abolish tumor cell growth as a new avenue of cancer therapy.

## Supporting Information

Figure S1 ***Tra2b*** **immunohistochemistry of the developing mouse brain.** Immunostaining of paraffin-embedded coronal sections at indicated developmental stages. KO but not controls or HET animals show ventriculomegaly of the third and lateral ventricles starting at around 14.5 dpc. Tra2b expression is effectively downregulated in KO brains compared to controls and HET animals. Cells of the ventricular and subventricular zones of the cortex show strongest decrease in staining intensity. Scale bar equals 400 µm; ctx, cortex; th, thalamus; cpt, caudoputamen; cp, cortical plate; iz, intermediate zone; svz, subventricular zone; vz, ventricular zone; 3v, third ventricle; lv, lateral ventricle; pc, choroid plexus.
(TIF)

Figure S2 **Analysis of putative splicing targets identified on mouse exon array.** Candidate exons were tested using isoform-specific RT-PCR. Each isoform was normalized to *Hprt.* The given percentages are expression changes of the respective isoform in the KO brain relative to a control brain. **(A)** Analysis of alternative splicing candidates that were identified as being differentially expressed as well (*Eomes, Kif11, Tcf4, Top2a*). Both isoforms of these transcripts show equal regulation into the same direction indicating transcriptional regulation. **(B)** Quantification of total *Eomes* and *Eomes* isoforms that include or exclude exon 4 showed coordinate downregulation, suggesting a general lower transcriptional expression but not alternative splicing of *Eomes* exon 4. **(C)** Candidates exclusively identified as being alternatively spliced (e.g. *Slu7*, *Nrg1*, *Sox6*). Slu7 and *Nrg1* are top-ranking splicing targets. Though, RT-PCR analyses show a coordinate downregulation of both isoforms arguing against any splicing related effect. For others there are only minor differences in isoform expression or one of two isoform is not detectable. *Eomes*, Eomesodermin; *Kif11,* kinesin family member 11; *Tcf4*, Transcription factor 4; *Top2a*, Topoisomerase 2a; *Slu7*, Slu7 splicing factor homolog; *Nrg1*, Neuregulin1; *Sox6*, *Sry* (sex determining region Y)-box 6; percentages show changes in control brains compared to KO brains for the respective isoform normalized to *Hprt*; error bars show the s.e.m.; significance levels are *p<0.05, **p<0.01, ***p<0.001 (Student’s t-test).
(TIF)

Dataset S1 **Candidate exons from mouse exon array analysis.** All exon data obtained from mouse exon array analysis were subjected to initial filtering omitting exons with less than ±1.5-fold up- or downregulation and p-values bigger than 0.05 yielding a list of 1,006 exons. Numerous transcripts had multiple exons detected as being alternatively spliced. Red font type indicates overlap with general changes in gene expression of the respective transcript. PSI[KOvsCTRL] describes the average changes of percentage splicing inclusion of a respective exon in 4 KO brains as compared to 4 CTRL brains based on the measured abundance of that exon on the arrays. The p-value was obtained by comparing single values of each genotype group using Student’s t-test. PROBESET_ID gives the Affymetrix probeset identification number as it is accessible and referred to in the online NetAffx database. EXON_LENGTH gives the length of the respective exon in base pairs. AGAA_COUNT gives the number of putative Tra2b binding sites included in the respective exon. AGAA_FREQ gives the relative frequency of Tra2b binding sites in an exon. The statistical probability of occurrence is once in 256 nucleotides and equals a frequency of 1. PROBESET_SEQ gives the sequence stretch that is covered by the respective probeset on the exon array. EXON_SEQ gives the sequence of the whole exon in coding 5′ to 3′ direction. The probeset region is shown in upper case.
(XLSX)

Dataset S2 **Differentially expressed candidate transcripts from mouse exon array analysis.** Data obtained from mouse exon array was analyzed with regard to general changes in gene expression. Initial filtering eliminated transcripts with less than ±1.5-fold up- or downregulation and p-values bigger than 0.05 yielding a list of 86 differentially expressed transcripts. TRANSCRIPT_ID gives the Affymetrix transcript identification number as it is accessible and referred to in the online NetAffx database. The p-value was obtained by comparing single values of gene expression level of each genotype group using Student’s t-test. F_C[KOvsCTRL] gives the relative fold-change of expression in KO mice as compared to CTRL brains.
(XLSX)

Dataset S3 **Filtered candidate exons from mouse exon array analysis.** All exon data obtained from mouse exon array analysis were filtered as stated in Fig. 4B yielding a total of 481 exons. PSI[KOvsCTRL] describes the average changes of percentage splicing inclusion of a respective exon in 4 KO brains as compared to 4 CTRL brains based on the measured abundance of that exon on the arrays. The p-value was obtained by comparing single values of each genotype group using Student’s t-test. PROBESET_ID gives the Affymetrix probeset identification number as it is accessible and referred to in the online NetAffx database. EXON_LENGTH gives the length of the respective exon in base pairs. AGAA_COUNT gives the number of putative Tra2b binding sites included in the respective exon. AGAA_FREQ gives the relative frequency of Tra2b binding sites in an exon. The statistical probability of occurrence is once in 256 nucleotides and equals a frequency of 1. PROBESET_SEQ gives the sequence stretch that is covered by the respective probeset on the exon array. EXON_SEQ gives the sequence of the whole exon in coding 5′ to 3′ direction. The probeset region is shown in upper case.
(XLSX)

Dataset S4 **Oligonucleotides and PCR conditions.** Amplicon indicates the target or purpose of the PCR and specifies the gene or the respective exon of interest. Forward and reverse oligonucleotides are both given in 5′ to 3′ direction. Product gives the length of the amplicon in base pairs. Platform describes the device or Polymerase kit used for the respective PCR. Oligonucleotide annealing temperatures are given in °C and end concentrations of MgCl_2_ in mM. Cycles indicates the number of repetitive PCR cycles. mm, mus musculus; hs, homo sapiens; E#, exon #; Δ#, isoform lacking exon #; fw, forward oligonucleotide; rev, reverse oligonucleotide; RT-PCR, semi-quantitative reverse transcription PCR; Roche LC, quantitative real-time PCR using Roche LC FastStart SYBRgreen; RTQ, quantitative real-time PCR.
(XLSX)
