# Supplementary figures and images for: Neuronal-Specific Deficiency of the Splicing Factor Tra2b Causes Apoptosis in Neurogenic Areas of the Developing Mouse Brain
Source: PLoS One. 2014 Feb 19;9(2):e89020. doi: 10.1371/journal.pone.0089020 (PMC3929626; doi:10.1371/journal.pone.0089020)

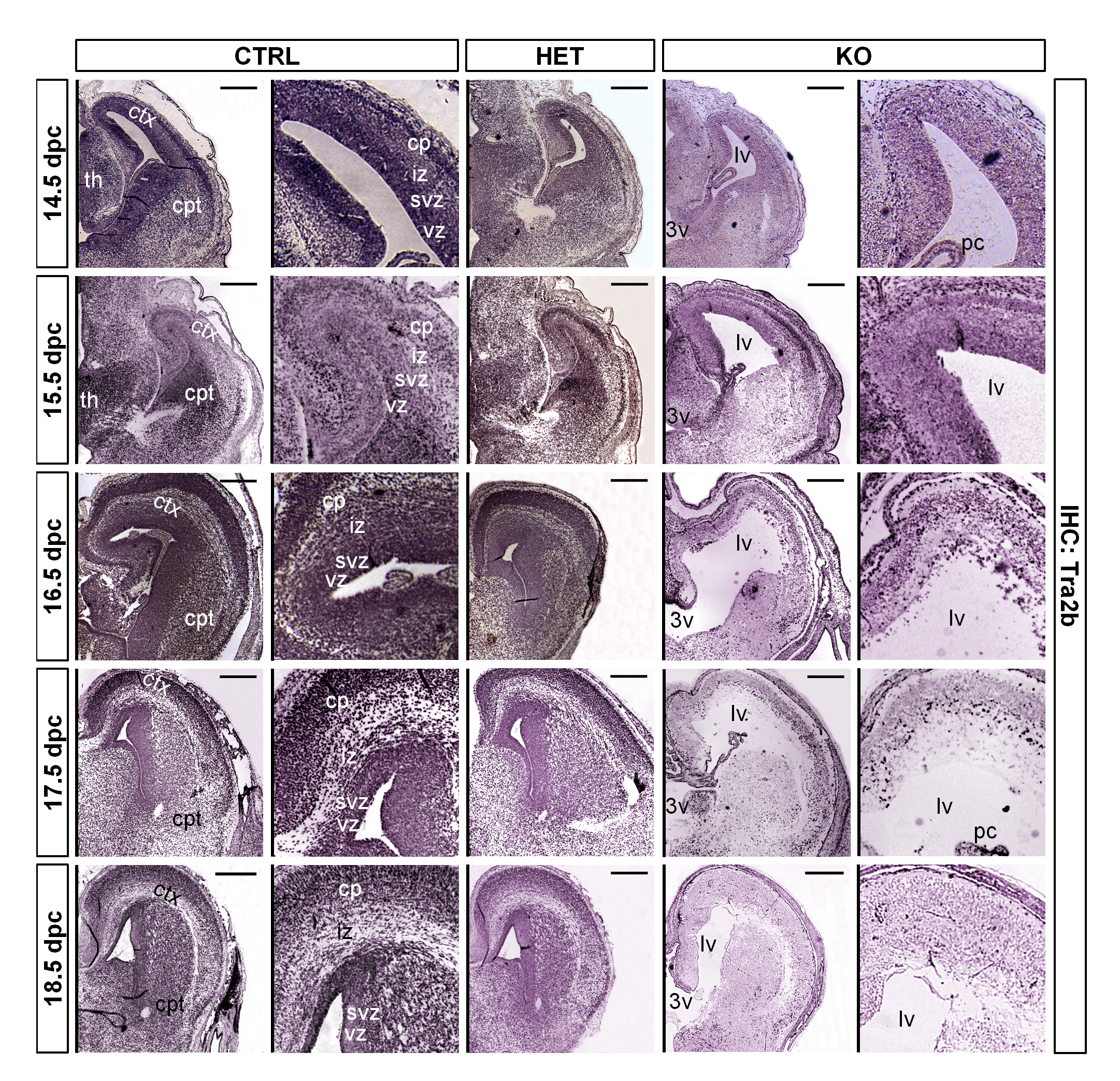

Supplement: Figure S1 — Tra2b immunohistochemistry of the developing mouse brain. Immunostaining of paraffin-embedded coronal sections at indicated developmental stages. KO but not controls or HET animals show ventriculomegaly of the third and lateral ventricles starting at around 14.5 dpc. Tra2b expression is effectively downregulated in KO brains compared to controls and HET animals. Cells of the ventricular and subventricular zones of the cortex show strongest decrease in staining intensity. Scale bar equals 400 µm; ctx, cortex; th, thalamus; cpt, caudoputamen; cp, cortical plate; iz, intermediate zone; svz, subventricular zone; vz, ventricular zone; 3v, third ventricle; lv, lateral ventricle; pc, choroid plexus. (TIF) [file pone.0089020.s001.tif]

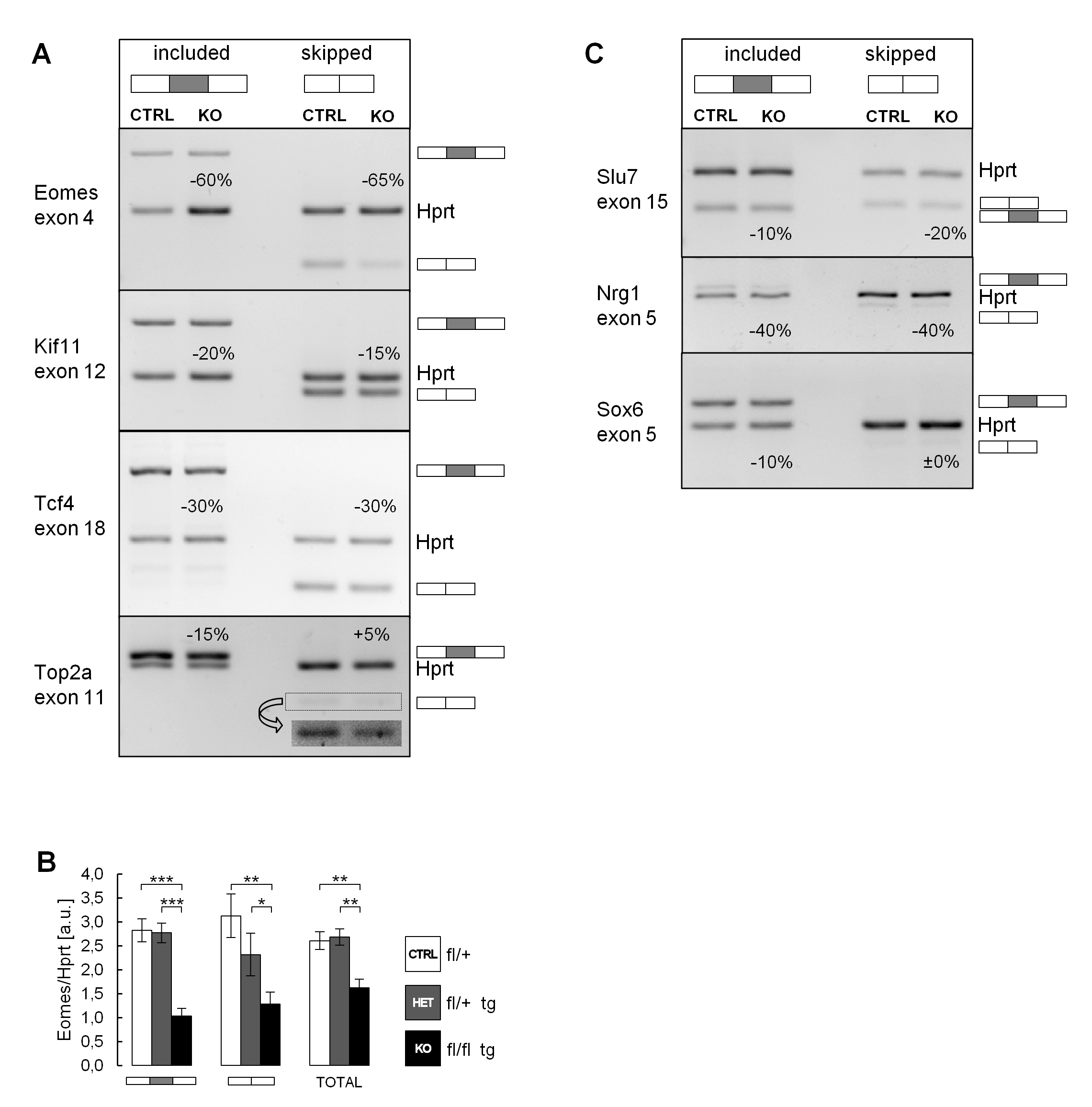

Supplement: Figure S2 — Analysis of putative splicing targets identified on mouse exon array. Candidate exons were tested using isoform-specific RT-PCR. Each isoform was normalized to Hprt. The given percentages are expression changes of the respective isoform in the KO brain relative to a control brain. (A) Analysis of alternative splicing candidates that were identified as being differentially expressed as well (Eomes, Kif11, Tcf4, Top2a). Both isoforms of these transcripts show equal regulation into the same direction indicating transcriptional regulation. (B) Quantification of total Eomes and Eomes isoforms that include or exclude exon 4 showed coordinate downregulation, suggesting a general lower transcriptional expression but not alternative splicing of Eomes exon 4. (C) Candidates exclusively identified as being alternatively spliced (e.g. Slu7, Nrg1, Sox6). Slu7 and Nrg1 are top-ranking splicing targets. Though, RT-PCR analyses show a coordinate downregulation of both isoforms arguing against any splicing related effect. For others there are only minor differences in isoform expression or one of two isoform is not detectable. Eomes, Eomesodermin; Kif11, kinesin family member 11; Tcf4, Transcription factor 4; Top2a, Topoisomerase 2a; Slu7, Slu7 splicing factor homolog; Nrg1, Neuregulin1; Sox6, Sry (sex determining region Y)-box 6; percentages show changes in control brains compared to KO brains for the respective isoform normalized to Hprt; error bars show the s.e.m.; significance levels are *p<0.05, **p<0.01, ***p<0.001 (Student’s t-test). (TIF) [file pone.0089020.s002.tif]
